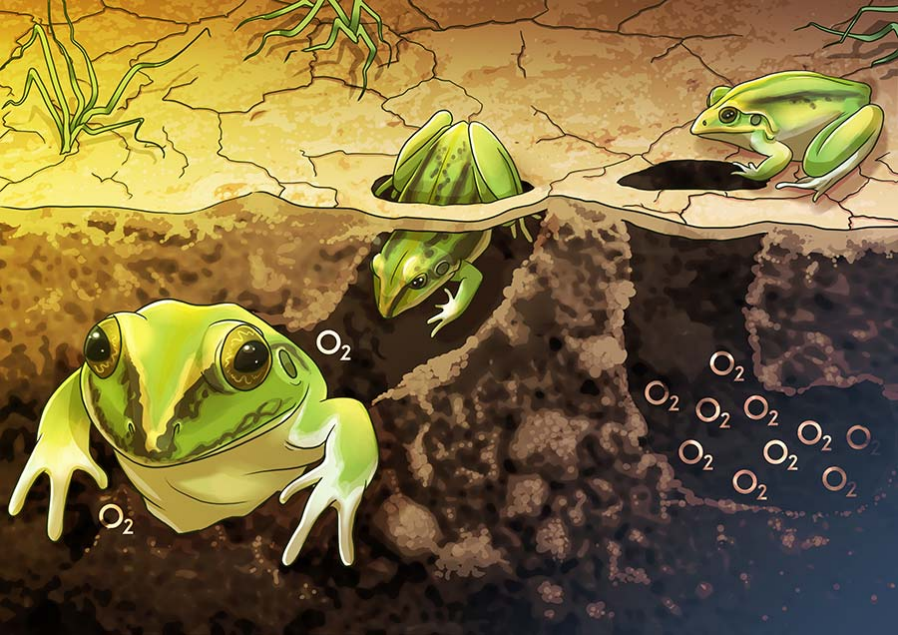# Sleeping beauties—how do frogs stay alive without oxygen?

**DOI:** 10.1093/conphys/coaa042

**Published:** 2020-04-30

**Authors:** Kim Birnie-Gauvin

**Affiliations:** Section for Freshwater Fisheries and Ecology, National Institute of Aquatic Resources, Technical University of Denmark Vejlsøvej 39, 8600 Silkeborg, Denmark

Most of us have heard of hibernation—the period where bears go dormant to escape harsh winter conditions. But, did you know that some frogs also undergo a form of hibernation? It is known as aestivation, and it is a period of dormancy associated with dry, summer conditions. Scientists think that aestivation evolved so animals could cope with challenges like limited food and water supplies. During aestivation, an animal dramatically decreases its metabolic rate for many months to conserve energy—this depression in metabolic rates may be the only way to stay alive.

Apparently, frogs can also reduce their metabolic rates for months on end, and Giulia Rossi and her team set out to determine how they do it. First, Rossi and her team examined the small holes in the wet, clay soil where green-striped burrowing frogs (*Cyclorana alboguttata*) tend to hang out during aestivation. The team found the burrows to be quite hypoxic, meaning extremely low in oxygen. So, the team built a tunnel where oxygen concentration would be high at one end and low at the other end to determine where the frogs spent most of their time during dry, summer conditions. It turns out that the frogs preferred the low oxygen end of the tunnel. But, how were they able to stay in low oxygen conditions for so long? And, why did they choose low oxygen when high oxygen conditions were still available?

To find out, Rossi and her team explored how low oxygen levels affect the frogs’ metabolism. They exposed the frogs to low oxygen or normal oxygen conditions for 7 weeks as the frogs entered aestivation. While we know from decades of research that most hibernating and aestivating animals lower their metabolic rate to conserve energy, we do not necessarily know how fast they do it. The team found that the frogs exposed to low oxygen conditions were able to lower their metabolic rate much more rapidly than the frogs maintained under normal oxygen conditions. Moreover, once frogs were aestivating, those that had been exposed to low oxygen had lowered their metabolism by nearly 30% more than the frogs that had been maintained under normal oxygen conditions. So, frogs actually choose microhabitats with low oxygen levels, like those small cavities in the soil, to help them slow down their metabolic rate faster and by more. Consequently, this allows these frogs to also slow down how fast their bodies are using stored resources and ultimately gives them better odds of surviving.

Usually, we think animals try to avoid hypoxic conditions. Yet, this study suggests that hypoxia-seeking behaviours are important for frogs during dormancy and that the actual microhabitats are important too. So, whether an organism hibernates or aestivates, it seems that being able to select microhabitats that are appropriate for dormancy may represent an adaptation key to the survival of species across the globe. These findings also suggest that some of these microhabitats, like a muddy burrow for example, which may seem unimportant to us humans, can actually represent the difference between life and death for some organisms. Indeed, these critical microhabitats may be of a higher priority for conservation than we may have previously understood. As global climate change, and particularly global warming persists, these periods of dormancy and associated habitats may become more and more important for the survival of an array of animals and their ecosystems.

It turns out that frogs are like sleeping beauty, but in small holes without oxygen…

Section Editor: Jodie L. Rummer

Illustration by Erin Walsh; Email: ewalsh.sci@gmail.com